# Comparison of Lower Limb Segments Kinematics in a Taekwondo Kick. An Approach to the Proximal to Distal Motion

**DOI:** 10.1515/hukin-2015-0060

**Published:** 2015-10-14

**Authors:** Isaac Estevan, Coral Falco, Julia Freedman Silvernail, Daniel Jandacka

**Affiliations:** 1 Department of Teaching of Music, Plastic and Corporal Expression. University of Valencia, Valencia, Spain.; 2 Department of Health Promotion and Development. University of Bergen, Bergen, Norway.; 3 Department of Kinesiology and Nutrition Sciences. University of Nevada Las Vegas, Las Vegas, USA.; 4 Department of Human Movemente Studies. Human Motion Diagnostic Center. University of Ostrava, Ostrava, Czech Republic.

**Keywords:** kinetic link, pattern, biomechanics, execution technique, combat sports

## Abstract

In taekwondo, there is a lack of consensus about how the kick sequence occurs. The aim of this study was to analyse the peak velocity (resultant and value in each plane) of lower limb segments (thigh, shank and foot), and the time to reach this peak velocity in the kicking lower limb during the execution of the roundhouse kick technique. Ten experienced taekwondo athletes (five males and five females; mean age of 25.3 ±5.1 years; mean experience of 12.9 ±5.3 years) participated voluntarily in this study performing consecutive kicking trials to a target located at their sternum height. Measurements for the kinematic analysis were performed using two 3D force plates and an eight camera motion capture system. The results showed that the proximal segment reached a lower peak velocity (resultant and in each plane) than distal segments (except the peak velocity in the frontal plane where the thigh and shank presented similar values), with the distal segment taking the longest to reach this peak velocity (p < 0.01). Also, at the instant every segment reached the peak velocity, the velocity of the distal segment was higher than the proximal one (p < 0.01). It provides evidence about the sequential movement of the kicking lower limb segments. In conclusion, during the roundhouse kick in taekwondo inter-segment motion seems to be based on a proximo-distal pattern.

## Introduction

Over the years, complex human movements such as kicking or throwing have been postulated as being performed in an open kinetic link pattern (Inamura and Jonshon, 2003; Lust et al., 2009; Pearson, 1997; Sujae and Koh, 2008; Zatsiersky, 1998). These types of movement patterns are analysed taking into account the specific characteristics of a kinetic link system, which means that the system has a fixed and more massive end (proximal segment), and a free and less massive end (distal segments) (Kreighbaum and Barthels, 1990; Putnam, 1993). A torque is applied to the proximal segment to initiate the system’s motion aiming to give the entire system angular momentum. Inter-segmental torques are internal to the system and, when applied, change the angular velocities of individual segments in the system. Thus, open kinetic link patterns are generally performed in a proximo-distal sequence characterised by building up the speed of the free distal end of the linked system by summing the individual velocities of the segments participating in the sequence (Putnam, 1991; 1993). It has been stated that the kick pattern is typically performed in this proximo-distal sequence: first, proximal segments accelerate while distal segments lag behind, and then proximal segments decelerate while distal segments accelerate (Sørensen et al., 1996). It often implies that each limb comes forward as the movement of its proximal segment reaches its greatest angular velocity with the distal end of the proximal segment having its greatest linear velocity at this time (Kreighbaum and Barthels, 1990). Thus, the ultimate velocity of the distal segment depends on the velocity of the proximal segment and the interactions of these segments (Lust et al., 2009). In taekwondo, kicks have often been described as a throwlike or swing movement (Serina and Lieu, 1991), however, up to date, there is a lack of consensus about how this proximo-distal sequence occurs.

Serina and Lieu (1991) categorized taekwondo kicks according to the trajectory as both swing or circular kicks and thrusts or linear kicks. The most frequent technique used in competition is the roundhouse kick (a circular kick) followed by linear kicks (Matsushigue et al., 2009). The roundhouse kick is the fastest kick in taekwondo (Pieter and Pieter, 1995). It starts with hip flexion in the sagittal plane followed by a knee extension while the thigh is rotating to kick the target in the lateral direction (Keum-Jae, 2005; Pearson, 1997). Traditionally, the scientific field has focused on linear kicks, yet, it has been recommended to study circular kicks, specifically the roundhouse kick (Falco et al., 2009). Some authors suggest that following traditional orientation regarding kicks, they are typically performed in a proximo-distal sequence, yet, they lack the empirical evidence to support this (Nien et al., 2006; Pearson, 1997). Others (i.e., Landeo et al., 2012; Sujae and Koh, 2008) postulate that kicks are not executed using this kind of sequence. According to Pearson (1997), traditionally in taekwondo, motion analyses have been limited to movements carried out in just one plane involving a two dimensional analysis. It is worth noting that despite a wide acknowledgement that the roundhouse kick is three dimensional in nature, relatively few three dimensional studies have been conducted and little kinematic data are available (Kim et al., 2011).

Thus, the purpose of this study was to analyse the resultant of the peak velocity of segments and the time to reach this peak velocity during the execution of the roundhouse kick technique in taekwondo. Authors hypothesized that the roundhouse kick was based on the proximo-distal sequence. Therefore, the proximal segments would acquire lower peak velocity than the distal end, and the distal end would reach this higher peak velocity last during the kick.

## Material and Methods

### Participants

Sample size estimation was carried out using the error measurement equation wherein data used came from previous studies (i.e., Estevan et al., 2013) with a proper sample determined of eight participants. Ten highly experienced (with at least eight years of experience (12.9 ±5.3 years) and training sessions three times per week minimum; some of them being World and/or National Champions), black belt taekwondo athletes (five males (28.6 ±2.7 years; 85.6 ±10.3 kg; 1.83 ±0.04 m) and five females (22.2 ±5.5 years; 59.6 ±7.7 kg; 1.66 ±0.07 m)) participated in this study. None of the athletes had a history of injury within the six months before data collection for this study. Before participating in the experiment, all participants gave their written informed consent.

### Measures

The participants stood with each foot on a force platform (Kistler 9286AA^®^, Switzerland). Ground reaction force data were sampled at 1200 Hz. Kinematic data during the roundhouse kick were synchronized with force data and collected at 240 Hz with an eight-camera motion capture system (Qualisys Oqus^®^, Sweden). Cameras were located surrounding the two force platforms with mutual offset by an angle of 45 degrees as a stance position (Estevan et al., 2013). The camera volume encompassed the area of the target. A light-emitting diode (LED) placed on the target generated the randomized signal to kick and was used to synchronize all the equipment (Estevan et al., 2012).

### Procedures

Twenty four retro-reflective markers were attached to the athletes’ body. Calibration markers were placed bilaterally on the lateral and medial malleolus, medial and lateral femoral epicondyles, greater trochanter of femur, and on the foot over the first and fifth metatarsal heads. The tracking markers were securely positioned to define the target, the trunk (acromion), the tenth thoracic vertebra, the chondral projection of the sternum, iliac spine, posterior superior iliac crest, and posterior calcaneus. Clusters with four tracking markers were placed on the thigh and shank and two other tracking markers were placed on the calcaneus. After a static calibration was recorded in the basic anatomical position, defining the segment dimensions and relationships between the calibration and tracking markers, the calibration markers were removed.

**Figure 1:**
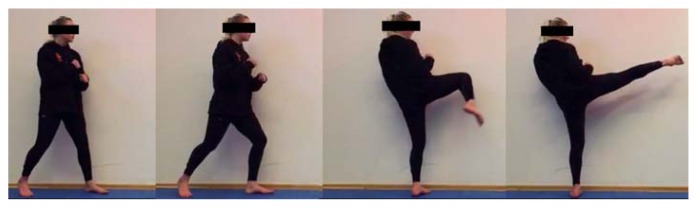
Images of a participant performing a roundhouse kick. From left to right: a stance; prior to the athlete’s kicking foot left the floor; the knee reaches the maximum flexion; impact.

Participants were allowed time for an individual warm-up. Throughout the test participant’s feet (barefoot) were positioned on the force plates with the kicking leg at the rear. Force plates were used to ensure relative symmetric loads in both feet (no trial was discarded due to asymmetries in the feet load) with a distribution of the resultant of the ground reaction force (% body weight) in the force plates of 53.56 ±4.99% in the front foot and 52.94 ±5.46% in the rear foot). Each participant’s preferred kicking distance was used as the target height (Gulledge and Dapena, 2008) and execution distance (Kim et al., 2010). The athletes received the standard instruction to kick the lateral side of the target “as fast as possible”. Each trial started when the LED was illuminated and athletes reacted by kicking the target (a surface of foam and nylon similar to a typical taekwondo handle pad (0.65 m of circumference) at the athletes’ chest height with the instep of the preferred lower limb (four athletes were right and six were leftfooted)). Each athlete performed 5 roundhouse kicks (Figure 1) with 2–3 min rest intervals between trials, after terminating each trial athletes returned to the force platforms. The protocol was approved by the University of Ostrava Ethics Committee and performed in accordance with the principles of the Declaration of Helsinki.

**Figure 2:**
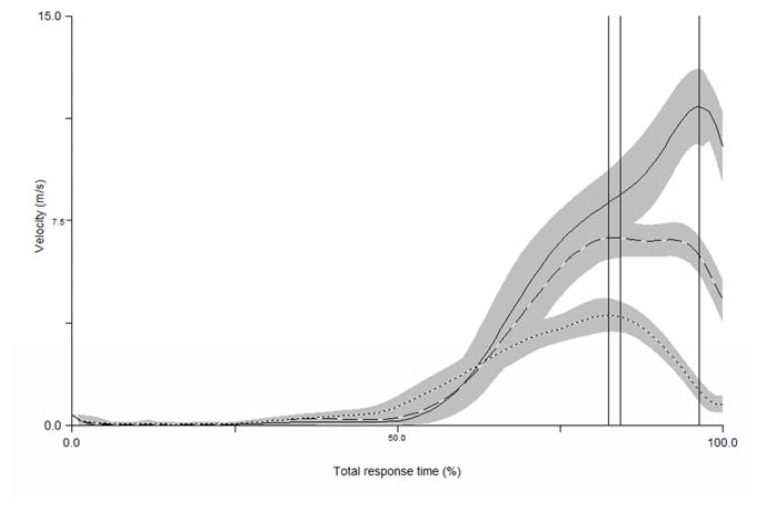
Mean of the resultant of velocity during the total response time in the roundhouse kick (n = 10). The dotted line represents the thigh segment, the dashed line represents the shank segment, and the solid line represents the foot. The solid area represents the standard deviation from each of the segments curve. Vertical solid lines from left to right inform about the timing of the thigh, shank and foot peak velocities.

The marker trajectory data were processed using Visual 3D software (C-motion^®^, USA). All extremity segments were modelled as a frustum of right circular cones, and the torso and pelvis were modelled as a cylinder. The three dimensional segment linear velocities were determined as the first derivative of the foot, shank and thigh centre of the gravity position with respect to the time. For the purpose of the study, we determined the velocity for each segment (i.e., value of the resultant as the root square of the sum of every square plane data and the value of the velocity in each plane) being the variables of the study as discrete, that is, the peak value of every segment velocity and the velocity of each segment when the others reached the peak value; and the time to reach the peak velocity. Finally, kinematic signals were normalized to 100% of the total response time (Figure 2).

### Statistical analysis

Statistical analyses were carried out using SPSS 20.0 (SPSS Inc., Chicago, IL). The preliminary analysis (Kolmogorov–Smirnov) showed a normal distribution of all the considered variables. The intra-class correlation coefficient (ICC) to test the reliability of the kinematic variables was calculated. Since no significant differences were found between lower limb kinematics of males and females (*p* > 0.05), statistical analyses of both genders were analyzed in conjunction to increase statistical power. Two consecutive one-way repeated measures analysis of variance (ANOVA) with “segment” (three levels: thigh, shank and foot) as within a subject factor were performed to calculate observed statistical power (*SP*) of the test and to compare kinematic variables (1^st^ to compare the time to peak velocity and the peak velocity (resultant and in every plane); 2^nd^ to compare velocity (resultant and in every plane) when every segment reached the peak velocity). Alpha was set at 0.05 for all statistical analyses. Significant factor effects and interactions were subsequently examined using the Bonferroni adjustment for multiple comparisons. Partial eta square (η_p_^2^) values below 0.01, 0.01–0.06, 0.06–0.14, and above 0.14 were considered to have trivial, small, medium, and large effect sizes, respectively (Cohen, 1988).

## Results

The ICC for the time to reach the peak velocity of the thigh was 0.99 (95% CI, 0.97–1.00), of the shank was 0.98 (95% CI, 0.92–1.00), and of the foot was 0.98 (95% CI, 0.91–1.00). The ICC for peak velocity (resultant and in every plane) ranged between 0.93 and 0.98 being the values for the thigh 0.98 (95% CI, 0.93–1.00), for the shank 0.98 (95% CI, 0.92–1.00), and for the foot 0.93 (95% CI, 0.76–0.99). This suggested that the data were reliable.

The first one-way repeated measures ANOVA revealed a main effect of the “segment” on each of the selected variables. That is, on time to reach the peak velocity (*F*_(1, 9)_ = 146.82; *p* < 0.001; η_p_^2^ = 0.94; *SP* = 1.00), resultant of the peak velocity (*F*_(1, 9)_ = 365.01; *p* < 0.001; η_p_^2^ = 0.98; *SP* = 1.00), peak velocity in the sagittal plane (*F*_(1. 9)_ = 116.20; *p* < 0.001; η_p_^2^ = 0.93; *SP* = 1.00), peak velocity in the frontal plane (*F*_(1, 9)_ = 74.67; *p* < 0.001; η_p_^2^ = 0.89; *SP* = 1.00), and peak velocity in the transverse plane (*F*_(1, 9)_ = 61.18; *p* < 0.001; η_p_^2^ = 0.87; *SP* = 1.00). The Bonferroni statistics adjusted for paired comparisons (Table 1) showed that the time to reach the peak velocity in the thigh and shank was lower than in the foot (*p* < 0.005 and *p* < 0.006, respectively).

**Figure 3:**
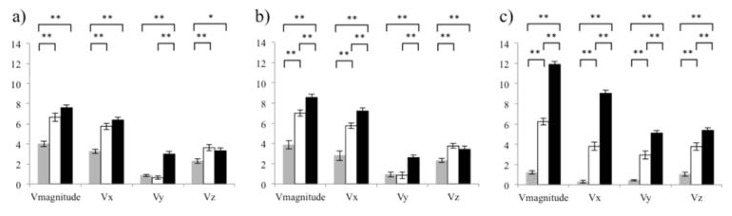
Histograms (group mean and SD) for every segment velocity (resultant and values in every plane) at the instant of peak velocity for the thigh (a), shank (b), and foot (c). The grey columns represent the thigh segment, the white columns represent the shank segment, and the black columns represent the foot. * and ** symbols over the segments values mean significant pairwise differences between these two segments (p < 0.02 and p < 0.01, respectively).

Moreover, the peak velocity of the thigh was lower than the peak velocity of the shank and foot (*p* < 0.001). Peak velocity of the shank was lower than the peak velocity of the foot (*p* < 0.001). Similar results were found in the sagittal and transverse planes, i.e., the peak velocity of the thigh in these planes was lower than the peak velocity of the shank and foot (*p* < 0.001). Furthermore, the peak velocity of the shank in the sagittal and transverse planes was lower than in the foot (*p* < 0.005). Finally, in the frontal plane, the peak velocity in the thigh and shank was lower than in the foot (*p* < 0.001). Figure 3 shows the results of the second one-way repeated measures ANOVA with regard to segments velocity (resultant and in every plane) when the thigh (a), shank (b) and foot (c) reached the peak velocity. Analysing the effect size of the two repeated measures ANOVA, with all the ηp2 values over 0.14, large effect in every of the differences were found.

## Discussion

Traditionally, research literature on combat sports has analysed execution techniques in relation to performance (Estevan et al., 2012, 2013; Gulledge and Dapena, 2008; Kim et al., 2011) pointing out that the majority of the techniques are characterised by great diversity and complexity of movements. In this line, scientific knowledge on proximo-distal sequence in taekwondo kicks is still limited (Kim et al., 2011). The present study aimed to analyse the resultant and components of the peak velocity of segments and the time to reach this peak in the roundhouse kick in order to verify the proximo-distal sequence in a sample of experienced taekwondo athletes. The ICC of the data for each of the variables analysed was close to 1.00, meaning there was a high reliability of the results. In response to the recommendation for biomechanical analysis (Smith et al., 2000), we analyzed empirical data of combat sport movement.

Authors who have analyzed coordination patterns in a kicking context (Kim et al., 2011) defined it as the relative movement pattern between interacting body parts during goal directed behavior. Although intralimb coordination was not analysed in this study, we compared the resultant of the peak velocity by segments in the kicking lower limb. The results of our study showed that the proximal segment (thigh) achieved lower peak velocity than the shank and the distal end (foot), while the foot segment reached the highest peak velocity. Moreover, this peak velocity was reached earlier by the thigh and shank than the foot, with the foot being the segment taking the longest to reach peak velocity (Figure 2). According to Sørensen et al. (1996), during the kick, proximal segments accelerate while distal segments lag behind. Later proximal segments decelerate while distal segments accelerate. This seems in line with other studies which have compared kick execution with the technique resulting in a whip-like action (Anderson and Sidaway, 1994), which is one of the main analogies that coaches suggest for improving taekwondo athletes’ technique.

The roundhouse kick is a multiplanar skill, with the kicking leg travelling in an arc towards the front with the knee in a chambered position (Falco et al., 2009). The knee is extended in a snapping movement, striking the target with the instep laterally (Keum-Jae, 2005). Pieter and Pieter (1995) pointed out the relevance of the lateral component (frontal plane) as one reason to generate the highest velocity in the roundhouse kick. In this line, the analysis of peak velocity in each of the planes (Table 1) showed that in the frontal plane even though the foot reached the highest peak velocity, the velocities of the thigh and shank were similar to each other. On the other hand, the sagittal and transverse components were similar to the results of the resultant value. That is, in these two planes the thigh peak velocity was lower than the shank and the foot. Furthermore, the shank peak velocity was lower than the foot. Thus, even though the kick pattern seems to be based on a proximo-distal one, athletes do not follow this strategy in all of the components of the movement.

In addition, in order to provide insight into the proximal to distal motion pattern in the roundhouse kick, the analysis of the velocity (the resultant and in every plane) when each of the three segments reached the resultant of the peak velocity was performed. In this line, across the three main instants, the foot velocity (resultant and three components) was higher than the thigh (Figure 3). Also, when the thigh reached its peak velocity (Figure 3a) the resultant, sagittal and transverse plane velocities of the shank were higher than those of the thigh. Nonetheless, at this instant, with the exception of the frontal plane, no differences between shank and foot velocities were found. Similar conditions existed when the shank reached peak velocity (Figure 3b); with the exception of the resultant and sagittal plane where the foot achieved higher velocity than the shank. Finally, when the foot reached its peak velocity, the velocity of the distal segment and the differences among segments during the kick were more plausible (Figure 3c); that is, the resultant of velocity in each plane of the foot achieved higher velocities than the shank and thigh, while the thigh seemed to decrease the velocity throughout. According to these results, it seems that the proximal to distal kick motion pattern is followed partially, whereas some planes do not follow the sequential movement. Although these results support previous studies that analysed the technique only in some planes (i.e., the sagittal plane (Landeo et al., 2012)), it should be noted that data in that study was only collected for the thigh and shank motion, but not for the foot motion. The analysis of the foot segment in the current study provided an insight regarding the differences in velocity with respect to the thigh and shank across the kick motion. Moreover, our results suggest that as the kick progresses, the differences among segments velocities increase, which could provide evidence of the mechanisms used to perform the kick based on this proximal to distal sequence (Sørensen et al., 1996; Putnam, 1993). In order to confirm and report thorough information about a kicking sequential pattern in every plane, future studies should assess movement coordination in taekwondo kicks.

Our results provide partial empirical support for the traditional view that kicks are typically performed in a proximo-distal sequence (Anderson and Sidaway, 1994; Nien et al., 2006; Pearson, 1997). This is indicated by the fact that the proximal segment reached a lower peak velocity than distal segments and this peak occurred earlier than that of the distal end. Thus, it could be stated that the roundhouse kick is based on a proximo-distal sequence in which athletes accelerate segments increasing the velocity progressively as the kick is developed, aiming to reach the highest peak velocity in the distal end of the linked system. These results could help coaches who often (without empirical information) focus technical training on starting kicks with the knee flexed, afterwards when athletes voluntarily flex their hip, the coach suggests extending the knee progressively but powerfully; being likely to use the phrase “whip-like action”.

Although a possible limitation of the current study is the small number of participants, an apriori estimation of the sample size indicated the need for only eight subjects to achieve 95% power of theoretical basis. It is also of note that the required level of athletes’ expertise limited the number of possible participants to attribute appropriate evidence in which these theoretical bases hinged on. Another limitation of our research relates to the initial stance position of the athletes prior to kicking. In our study, the participants had a diagonal initial stance which was characterized by maintaining one foot in front of the other with the body positioned laterally to the target and both feet approximately diagonally to the target. Since some previous studies in the taekwondo field (Estevan et al., 2013) stated that the initial stance position prior to kicking was a factor that affected performance, future studies should analyze the effect of the stance position in the execution technique or inter-segment motion patterns during kicks in taekwondo.

## Conclusion

Sport sciences research in combat sports is quite recent. Research supporting theoretical explanations of technical executions is scarce. Thus, it is common to see coaches who try to explain athletes’ performance without a theoretical basis. This study provides empirical knowledge that partially supports inter-segment motion based on a proximo-distal pattern during the roundhouse kick in taekwondo i.e., the proximal segment reaches a lower peak velocity than distal segments, with the distal segment reaching the highest peak velocity last. This movement pattern could support traditional coaches’ instructions based on their technical expertise related to inter-segment motion patterns. The use of the proximal to distal motion pattern as a basis for coaching interventions seems appropriate.

## Figures and Tables

**Table 1:** Descriptive data of the time to peak velocity and the peak velocity of segments in the roundhouse kick in taekwondo

	Thigh		Shank		Foot	

	M	SD	M	SD	M	SD
T peak V (s)	0.604	0.092 ^a^	0.620	0.108 ^b^	0.705	0.100 ^ab^
Peak V (m/s)	4.00	0.58 ^ab^	7.02	0.65 ^ac^	11.90	1.38 ^bc^
Peak Vx (m/s)	3.26	0.43 ^ab^	5.77	0.57 ^ac^	9.04	1.61 ^bc^
Peak Vy (m/s)	0.85	0.22 ^a^	0.87	0.67 ^b^	5.11	1.47 ^ab^
Peak Vz (m/s)	2.28	0.43 ^ab^	3.76	0.49 ^ac^	5.38	1.29 ^bc^

T peak V: time to the peak velocity in each segment in seconds (s).

Peak V: resultant of the peak velocity of each segment in m/s; Vx, Vy and Vz refer to the velocity in each of the three planes: sagittal, frontal and transverse, respectively.

Similar letter (^a, b or c^) to the right of the mean and standard deviation in every variable means significant differences in the pairwise comparisons (p < 0.01).
